# Artificial Intelligence vs. Human Experts in Temporomandibular Joint MRI Interpretation: A Systematic Review

**DOI:** 10.3390/healthcare14081066

**Published:** 2026-04-17

**Authors:** Marijus Leketas, Inesa Stonkutė, Miglė Miškinytė, Dominykas Afanasjevas

**Affiliations:** 1 Faculty of Odontology, Medical Academy, Lithuanian University of Health Sciences, J. Lukšos-Daumanto 2, LT-50106 Kaunas, Lithuania; marijus.leketas@lsmu.lt; 2 Department of Maxillofacial Surgery, Medical Academy, Hospital of Lithuanian University of Health Sciences, Eiveniu 2, LT-50161 Kaunas, Lithuania; migle.miskinyte@stud.lsmu.lt (M.M.); dominykas.afanasjevas@stud.lsmu.lt (D.A.)

**Keywords:** temporomandibular joint (TMJ), temporomandibular disorders (TMDs), magnetic resonance imaging (MRI), artificial intelligence (AI), anterior disc displacement, osteoarthritis, joint effusion, disc perforation

## Abstract

**Background**: Magnetic resonance imaging (MRI) is the reference standard for evaluating temporomandibular joint (TMJ) disorders, particularly for assessing disc position, joint effusion, and degenerative changes. With increasing imaging demands and advances in deep learning, artificial intelligence (AI) has emerged as a potential adjunct to expert interpretation. This systematic review aimed to compare the diagnostic performance of AI-based models with that of human experts in TMJ MRI analysis. **Methods**: This review was conducted in accordance with the PRISMA 2020 guidelines and prospectively registered in PROSPERO (CRD420251174127). A systematic search of PubMed/MEDLINE, ScienceDirect, Wiley Online Library, and Springer Nature Link was performed for studies published between 2020 and 2026. Eligible studies included human participants undergoing TMJ MRI and evaluated AI, machine learning, or deep learning models against human expert interpretation. Extracted outcomes included sensitivity, specificity, accuracy, area under the receiver operating characteristic curve (AUC), and agreement metrics. Risk of bias was assessed using QUADAS-2. Due to substantial heterogeneity, a narrative synthesis was conducted. **Results**: Five retrospective diagnostic accuracy studies were included, comprising sample sizes ranging from 118 to 1474 patients. Target conditions included anterior disc displacement, joint effusion, osteoarthritis, and disc perforation. AI models demonstrated strong discriminative performance, with reported AUC values ranging from 0.79 to 0.98. In direct comparisons, AI achieved diagnostic accuracy comparable to experienced radiologists. AI systems frequently demonstrated higher specificity and similar overall accuracy, whereas human experts often showed higher sensitivity. In osteoarthritis assessment, AI performance approached expert level and exceeded that of less experienced readers. All studies were retrospective and predominantly single-center, with heterogeneous reference standards and limited external validation. **Conclusions**: AI achieves diagnostic performance comparable to experienced clinicians in TMJ MRI interpretation and shows promise as a decision-support tool. Nevertheless, it should be regarded as complementary to, rather than a replacement for, expert radiological assessment pending further rigorous validation.

## 1. Introduction

The temporomandibular joint (TMJ) is a bilateral synovial articulation that connects the mandibular condyle with the mandibular fossa and articular eminence of the temporal bone [1]. It contains an interposed fibrocartilaginous disc that divides the joint into superior and inferior compartments, permitting both rotational (hinge) and translational (gliding) movements [2]. Owing to its complex anatomy and biomechanics, the TMJ is one of the most active joints in the human body and is particularly susceptible to functional disorders and structural pathology.

Temporomandibular disorders (TMDs) are a heterogeneous group of musculoskeletal and neuromuscular conditions affecting the TMJ complex, the masticatory muscles, and associated bony and soft tissue structures [3]. They are among the most common chronic musculoskeletal diseases of the orofacial region, second only to low back pain worldwide, with a prevalence of approximately 31% in adults and up to 11% in children and adolescents, showing a higher incidence in women and a peak between 20 and 45 years of age. TMDs have a multifactorial etiology, encompassing biomechanical, inflammatory, psychological, genetic, and environmental factors, and are frequently associated with systemic comorbidities such as osteoarthritis, cardiovascular disease, and thyroid dysfunction [4]. Clinically, they manifest as pain and noise in the TMJ or masticatory muscles, and restricted or asymmetric mandibular movement, and may extend to ear-related symptoms, headaches, or cervical pain [5]. In persistent cases, TMDs can lead to degenerative joint changes, occlusal disturbances, facial asymmetry, and significant psychosocial impact, including depression, anxiety, and sleep disruption [6].

Magnetic resonance imaging (MRI) is widely regarded as the gold standard for TMJ disorder evaluation because of its superior ability to visualize soft tissues, including the articular disc, masticatory muscles, and surrounding structures, without the use of ionizing radiation [7]. Compared with other imaging modalities, MRI provides excellent spatial and contrast resolution, making it particularly effective in assessing disc–condyle relationships, joint effusion, and inflammatory or degenerative changes [8]. This detailed visualization allows clinicians to detect internal derangements, such as disc displacement, which is the most prevalent TMD [7]. While the diagnosis of TMDs often relies on history and physical examination, MRI offers crucial complementary information when clinical findings are ambiguous, and recent advances in artificial intelligence are further enhancing its diagnostic utility [9].

Interpretation of TMJ MRI is primarily performed by radiologists and oral and maxillofacial radiologists, whose expertise is essential for accurate diagnosis and clinical decision-making. These specialists evaluate disc morphology and position, condylar and articular eminence morphology, joint effusion, and signs of degenerative or inflammatory change. Importantly, radiologists integrate imaging findings with clinical history and functional assessment, enabling differentiation between physiological variations and pathological conditions [1]. However, TMJ MRI interpretation may be subject to inter-observer variability, particularly among less experienced readers, and can be time-consuming in high-volume clinical settings. Consequently, radiologists serve as the clinical benchmark against which emerging AI-based diagnostic systems are evaluated [2,8].

Artificial intelligence has recently been applied in the field of TMJ imaging, particularly MRI, with the aim of improving diagnostic support. Convolutional neural networks and related deep learning methods have been tested for detecting internal derangements such as disc displacement and for segmenting joint structures, showing potential as complementary tools to human expertise [2,10,11]. The growing body of literature highlights AI’s ability to process complex imaging data and assist in clinical decision-making, although its performance compared to trained radiologists remains an area of active research. Therefore, the present study aimed to directly compare AI performance with human assessment in TMJ MRI interpretation.

## 2. Materials and Methods

### 2.1. Protocol and Registration

This systematic review was conducted in accordance with the Preferred Reporting Items for Systematic Reviews and Meta-Analyses (PRISMA) 2020 guidelines [12]. The protocol was prospectively registered with the International Prospective Register of Systematic Reviews (PROSPERO; registration ID: CRD420251174127). The research question was formulated using the PICO framework presented in Table 1.

### 2.2. Eligibility Criteria

#### 2.2.1. Inclusion Criteria

Studies were included if they met the following conditions:

1. Involved human participants undergoing temporomandibular joint (TMJ) magnetic resonance imaging (MRI) for any indication.
2. Applied artificial intelligence (AI), machine learning (ML), or deep learning (DL) methods for image analysis, interpretation, classification, segmentation, or diagnostic decision support.
3. Included diagnostic assessment by human experts, including radiologists, oral and maxillofacial radiologists, or oral/maxillofacial surgeons.
4. Reported at least one diagnostic or performance outcome, such as sensitivity, specificity, accuracy, AUC, Dice coefficient, IoU, image quality improvement, or clinical decision-support metrics.
5. Were designed as original research studies, including prospective or retrospective diagnostic accuracy studies, cohort studies, or clinical trials.
6. Published in the English language with full-text availability.
7. Published between 2020 and 2026.

#### 2.2.2. Exclusion Criteria

Studies were excluded if they met any of the following conditions:

1. Involved non-human subjects, such as animals or in vitro studies.
2. Did not include AI, ML, or DL methods in TMJ MRI analysis.
3. Were non-original publications, such as reviews, editorials, case reports, letters, or conference abstracts without full text.
4. Lacked sufficient data to extract or calculate diagnostic or performance outcomes.

### 2.3. Information Sources and Search Strategy

A comprehensive literature search was conducted between 21 October 2025 and 21 January 2026, to identify relevant studies investigating the application of artificial intelligence (AI) in temporomandibular joint (TMJ) magnetic resonance imaging (MRI). The following electronic databases were systematically searched: PubMed/MEDLINE, ScienceDirect, Wiley Online Library, and Springer Nature Link. The search was restricted to human studies published in English between January 2020 and January 2026.

In accordance with PRISMA 2020 guidelines, a systematic search strategy was developed using Boolean operators. The primary search terms were combined as follows:

(Temporomandibular Joint OR TMJ) AND (Magnetic Resonance Imaging OR MRI) AND (Artificial Intelligence OR Machine Learning OR Deep Learning OR Neural Network OR Convolutional Neural Network OR CNN OR Computer-Aided Diagnosis OR CAD).

The same Boolean framework was applied consistently across all databases, with minor syntax adjustments as required by individual database interfaces. The complete search strategy is provided in Supplementary Table S1.

All retrieved records were imported into EndNote reference management software (version 21, Clarivate, London, UK), where duplicate entries were identified and removed prior to screening. In addition, the reference lists of all included articles were manually reviewed to identify any further relevant studies.

### 2.4. Study Selection

The study selection process was conducted independently by three reviewers (I.S., M.M., and D.A.) under the supervision of a senior investigator (M.L.). The selection proceeded in three phases: initial title screening to assess topic relevance, abstract screening to evaluate compliance with the predefined eligibility criteria, and full-text review to determine final inclusion. Any disagreements between reviewers were resolved through discussion and consensus, with a fourth reviewer consulted when necessary. The entire selection process was summarized and illustrated using a PRISMA flow diagram, detailing the number of records identified, screened, excluded, and included in the final analysis.

### 2.5. Data Extraction

Three reviewers (I.S., M.M., and D.A.) independently extracted data using a standardized extraction form developed and piloted on three studies. Extracted variables included study characteristics, patient demographics, MRI acquisition parameters, AI architecture and training approaches, comparator expertise levels, diagnostic performance metrics, and study limitations. Disagreements were resolved through discussion; if consensus could not be reached, a senior reviewer (M.L.) adjudicated the decision.

### 2.6. Data Items

Data obtained from the included articles were organized into the following categories:

Author (Year): Identifies the study’s primary author and year of publication.

Study Design: Specifies the methodological design of the study (e.g., retrospective, prospective, diagnostic accuracy study).

Sample Size: Indicates the total number of participants or images analyzed.

Patient Characteristics: Summarizes key demographic or clinical information related to the study population.

MRI Protocol: Describes imaging parameters, field strength, and acquisition techniques used for TMJ MRI.

AI Methodology: Details the artificial intelligence approach, including algorithm/model type, training data, validation strategy, and analysis pipeline.

Comparator: Identifies the reference standard used, such as expert radiological assessment or manual segmentation.

Outcome Measures: Lists the main diagnostic and performance metrics reported (e.g., sensitivity, specificity, accuracy, AUC, Dice coefficient, IoU).

Key Findings: Summarizes the main results and conclusions relevant to AI performance or clinical utility.

### 2.7. Risk of Bias Assessment

The risk of bias of the included diagnostic accuracy studies was assessed using the Quality Assessment of Diagnostic Accuracy Studies 2 (QUADAS-2) tool [13]. QUADAS-2 evaluates four domains: (1) patient selection, (2) index test, (3) reference standard, and (4) flow and timing. For each domain, risk of bias is judged as low, high, or unclear based on signaling questions. In addition, applicability concerns are assessed for the first three domains (patient selection, index test, and reference standard).

For this review, QUADAS-2 was applied with additional signaling considerations relevant to AI-based diagnostic models in TMJ MRI:

- Patient selection: sampling method (consecutive vs. convenience), inclusion/exclusion criteria, enrichment for specific TMD subgroups (e.g., surgical or CT-referred cohorts).
- Index test (AI model): whether the AI model was pre-specified, whether the index test was interpreted without knowledge of the reference standard, and (for AI-specific adaptation) whether training, validation, and test sets were clearly separated at the patient level to minimize data leakage.
- Reference standard: appropriateness of the reference standard for the target condition (e.g., surgery or CT for structural changes, expert MRI interpretation for functional changes) and blinding to the index test.
- Flow and timing: interval between index test and reference standard, consistency of reference standard across participants, and completeness of follow-up.

Three reviewers (I.S., M.M., and D.A.) independently assessed all included studies using the QUADAS-2 tool, applying a piloted and standardized evaluation form. Discrepancies in domain-level judgments were resolved through discussion; when consensus could not be achieved, a senior reviewer (M.L.) made the final determination. Domain-level assessments are presented in tabular format, with a corresponding narrative synthesis of risk of bias and applicability concerns reported in Section 3.

### 2.8. Effect Measures

Because the review focuses on diagnostic performance of AI versus human experts for TMJ MRI interpretation, the primary effect measures extracted included the following:

- Sensitivity;
- Specificity;
- Accuracy;
- Area under the receiver operating characteristic curve (AUC/AUROC);
- Dice coefficient and intersection-over-union (IoU) for segmentation tasks;
- Cohen’s kappa or intraclass correlation coefficient (ICC) for agreement measurements;
- Positive predictive value (PPV) and negative predictive value (NPV), when reported.

All effect measures were extracted as reported in the individual studies without transformation.

### 2.9. Synthesis Methods

Due to substantial clinical, methodological, and outcome heterogeneity across included studies, a quantitative meta-analysis was not planned and was not conducted. Heterogeneity arose from differences in diagnostic targets (anterior disc displacement, joint effusion, osteoarthritis, and disc perforation), AI model architectures, training and validation strategies, MRI protocols and sequences, units of analysis (per joint, per condyle, or per image), outcome definitions, and human comparator groups. In addition, studies reported performance metrics using heterogeneous formats (e.g., accuracy, sensitivity/specificity at study-specific thresholds, AUROC values, multiclass outputs), without providing comparable effect sizes with associated variance. As a result, statistical pooling and formal heterogeneity estimation (e.g., Q statistic or I^2^) were not methodologically appropriate. The limited number of studies per diagnostic category precluded the application of hierarchical diagnostic meta-analytic models.

Accordingly, a structured narrative synthesis was performed. Studies were organized according to clinical target condition (disc displacement, effusion, osteoarthritis, disc perforation), type of AI methodology (e.g., convolutional neural networks, ensemble models, explainable AI frameworks), and human comparator category (e.g., radiologists, oral and maxillofacial surgeons, other expert clinicians).

The narrative synthesis summarizes the following:

- Study characteristics and key methodological features;
- MRI acquisition protocols and data preprocessing approaches;
- AI model architecture, training procedures, validation strategy, and transparency or explainability features;
- Diagnostic performance of AI models relative to human experts;
- Strengths, limitations, and potential sources of variation across studies.

Where numerical outcomes were available, results were presented descriptively in structured summary tables, without statistical pooling, to facilitate transparent comparison while avoiding inappropriate quantitative synthesis.

### 2.10. Reporting Bias Assessment

Because no meta-analysis was performed and the number of eligible studies is expected to be limited, formal statistical assessment of reporting bias (e.g., funnel plot asymmetry) was not applicable.

Instead, potential reporting bias will be evaluated qualitatively by the following:

- Comparing outcomes described in the methods with those reported in the results;
- Inspecting selective reporting of AI performance metrics;
- Assessing whether studies omitted adverse or poor-performing models;
- Reviewing completeness of reporting for training/test data splits and validation methods.

Any suspected reporting bias will be documented narratively.

### 2.11. Certainty of Evidence Assessment

Certainty of evidence was evaluated using the Grading of Recommendations Assessment, Development and Evaluation (GRADE) framework adapted for diagnostic test accuracy studies [14]. For each clinical outcome (joint effusion, anterior disc displacement, osteoarthritis, and disc perforation), evidence was initially considered high certainty and downgraded as appropriate.

The following domains were assessed:

- Risk of bias, informed by QUADAS-2 judgments;
- Inconsistency, based on variability of diagnostic performance across studies;
- Indirectness, considering applicability of populations, MRI protocols, AI models, and reference standards;
- Imprecision, reflecting sample size, number of studies per outcome, and absence of pooled estimates;
- Publication bias, evaluated qualitatively due to lack of meta-analysis.

## 3. Results

### 3.1. Results of Study Selection

**Figure 1:**
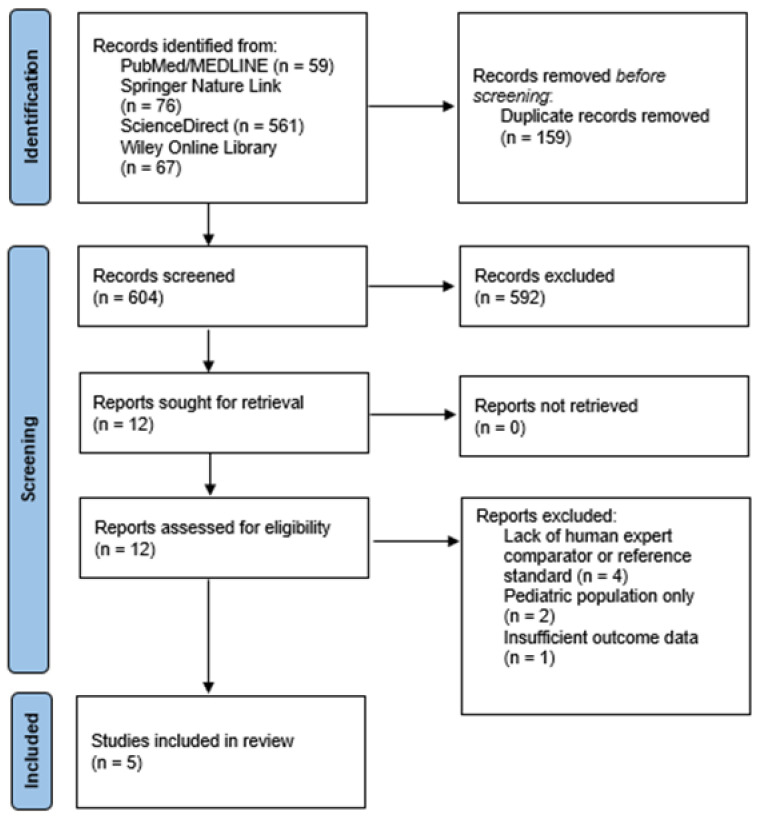
PRISMA flow diagram of study selection.

The search yielded 763 citations. After removing 159 duplicates, 604 records underwent title/abstract screening, and 12 full texts were assessed for eligibility. Five studies met all inclusion criteria and were included in the qualitative synthesis [6,10,15,16,17]. These studies evaluated AI or machine-learning systems for interpreting TMJ MRI in the diagnosis of anterior disc displacement, joint effusion, osteoarthritis, or disc perforation. The PRISMA flow diagram is provided in Figure 1.

### 3.2. Study Characteristics

Key characteristics of the included studies are summarized in Table 1. All five studies were retrospective diagnostic-accuracy investigations conducted in East Asia between 2021 and 2025. The sample sizes ranged from 118 patients [16] to 1474 patients [6]. Clinical targets included the following:

- Anterior disc displacement (ADD) [10,15];
- TMJ effusion [6];
- TMJ osteoarthritis [16];
- Disc perforation [17].

MRI examinations were performed on 1.5T or 3.0T scanners, typically using sagittal oblique PD- and T2-weighted sequences. All studies implemented machine-learning or deep-learning models, mostly convolutional neural networks (CNNs) trained for classification. Human comparators included radiologists, oral-maxillofacial specialists, or, for surgical outcomes, intraoperative confirmation.

### 3.3. Risk of Bias (QUADAS-2)

**Figure 2:**
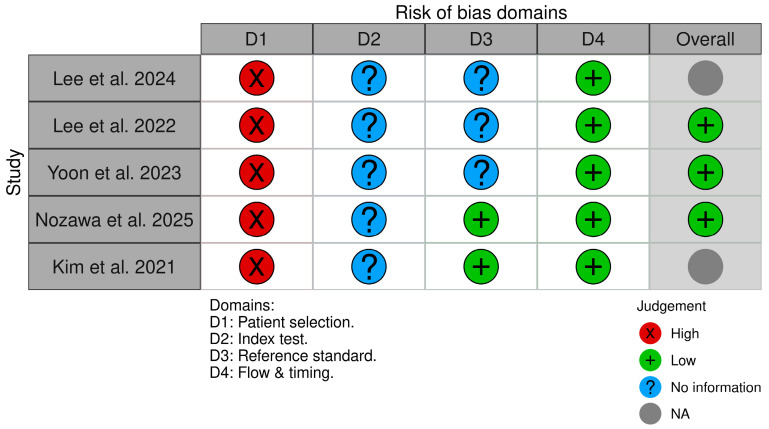
QUADAS-2 risk-of-bias traffic-light plot showing domain-level judgments (patient selection, index test, reference standard, flow and timing) for each included study [6,10,15,16,17].

Risk of bias and applicability concerns assessed using QUADAS-2 are summarized in Table 2 and visually presented in Figure 2. Overall, all five studies were judged to have a high risk of bias in the patient selection domain, largely because they were retrospective single-center investigations and included potentially disease-enriched cohorts. The index test domain was rated as unclear risk across studies because reporting of AI validation procedures—particularly patient-level dataset separation, methods to prevent data leakage, hyperparameter tuning, and blinding—was insufficient to exclude methodological bias. With respect to the reference standard, risk of bias was considered low in studies using CT-based osteoarthritis diagnosis [16] or intraoperative confirmation [17], whereas it remained unclear in studies relying solely on expert MRI interpretation without explicit reporting of blinding procedures [6,10,15]. The flow and timing domain was rated low risk for all studies. Applicability concerns were low to moderate, reflecting clinically relevant MRI protocols but restricted generalizability due to single-institution populations. A visual summary of the QUADAS-2 domain judgements is presented in Table 3.

### 3.4. AI Validation Characteristics

A structured assessment of AI validation procedures was conducted across the included studies. All five studies reported dataset partitioning into training and testing sets. Lee et al. (2022) [10] and Lee et al. (2024) [6] used temporal splitting, whereby earlier patient cohorts were used for model training and later cohorts for evaluation. Yoon et al. (2023) [15] implemented internal validation and additionally performed independent external validation using data from a separate institution. Nozawa et al. (2025) [16] applied 5-fold cross-validation. Kim et al. (2021) [17] described model training and validation procedures based on extracted MRI features. However, explicit clarification of patient-level separation and detailed data leakage prevention safeguards were generally not provided. Only one study incorporated independent external validation. This limited reporting of methodological safeguards contributed to uncertainty regarding model generalizability and informed the QUADAS-2 index test judgments and overall certainty assessment.

### 3.5. Results of Individual Studies

A comparative overview of MRI acquisition characteristics (including field strength) and diagnostic performance for each included study is provided in Table 4.

#### 3.5.1. Anterior Disc Displacement (ADD)

In Lee et al. (2022) [10], a VGG-based CNN was trained on 3.0T sagittal oblique TMJ MRI and achieved an AUC of approximately 0.88 with an accuracy of 0.77 using a fine-tuned model. Performance improved with an ensemble approach, increasing accuracy to 0.83 and sensitivity to 0.82, with a concomitant reduction in specificity to 0.85. In this study, AI demonstrated substantially higher specificity (by more than 10%) than both experts, while experts showed higher sensitivity; overall diagnostic accuracy did not differ significantly between AI and human readers on statistical testing (Table 4) [10]. In Yoon et al. (2023) [15], a two-stage explainable deep learning model was evaluated using 1.5T TMJ MRI for internal testing and 3.0T MRI for external validation, achieving an AUROC of 0.985 internally and 0.960 externally, with sensitivity 0.926–0.950 and specificity 0.892–0.919. Although expert interpretations defined the reference standard, clinician performance metrics were not consistently reported in parallel with AI outputs, limiting strict numerical head-to-head comparison despite robust external validation across scanners and centers (Table 4) [15].

#### 3.5.2. TMJ Effusion

Lee et al. (2024) [6] provided the most direct AI-versus-human comparison and used a 3.0T MRI protocol incorporating PD- and T2-weighted images (Table 4). The VGG16-based model achieved an AUC of 0.7895, with specificity 87.25% and sensitivity 57.43%. Using an ensemble configuration, AI achieved an accuracy of 74.21%, exceeding the reported human expert accuracy of 67.71%. As summarized in Table 4, this pattern suggests that AI improved overall accuracy primarily through higher specificity (fewer false positives), whereas human experts demonstrated higher sensitivity [6].

#### 3.5.3. TMJ Osteoarthritis

In Nozawa et al. (2025) [16], deep learning models were evaluated for MRI-based osteoarthritis classification using mixed 1.5T and 3.0T MRI protocols, with CT-based DC/TMD criteria as the reference standard (Table 4). The best-performing architecture (ResNet18) achieved an AUC of 0.91–0.93 and accuracy of 0.85–0.88, with excellent agreement against CT classification (κ = 0.95). Importantly, the study included a reader comparison: expert performance was reported at AUC 0.94 for at least one experienced oral radiologist, while residents performed substantially worse (Table 4). Overall, AI performance approached expert level and exceeded resident performance, supporting its potential value for decision support and reduction of inter-reader variability [16].

#### 3.5.4. Disc Perforation

Kim et al. (2021) evaluated disc perforation prediction using 3.0 T MRI with intraoperative surgical confirmation as the reference standard (Table 4) [17]. The multilayer perceptron achieved an AUC of 0.940, while the random forest achieved an AUC of 0.918, with sensitivity up to 96.3% [18]. Because perforation status was verified surgically, these results are best interpreted as AI performance against a definitive gold standard, however, the study did not report quantified expert-reader diagnostic performance relative to surgery, thereby limiting direct AI-human comparison (Table 4) [17].

### 3.6. Results of Syntheses

A quantitative meta-analysis was not performed due to substantial clinical and methodological heterogeneity among the included studies. First, the units of analysis varied considerably, with some studies reporting outcomes at the patient level, while others analyzed individual temporomandibular joints or condyles, thereby limiting direct comparability of diagnostic performance metrics. Second, the studies addressed different target pathologies, including anterior disc displacement, joint effusion, osteoarthritis, and disc perforation, each representing distinct diagnostic and clinical tasks.

Further heterogeneity arose from the use of diverse reference standards, ranging from expert MRI interpretation to CT-based diagnostic criteria and intraoperative surgical confirmation, resulting in variability in the definition of ground truth. Additionally, substantial differences existed in AI methodologies, including model architectures (e.g., convolutional neural networks and traditional machine learning algorithms), data preprocessing techniques, and validation strategies (internal versus external validation). Variations in diagnostic thresholds and reported outcome measures (e.g., AUC, accuracy, sensitivity, specificity, and kappa statistics) further complicated cross-study comparisons.

Importantly, several studies did not provide sufficient data, such as confusion matrices or 2 × 2 contingency tables, precluding the calculation of pooled sensitivity and specificity required for meta-analytic synthesis. Differences in study populations, with some investigations including general temporomandibular disorder cohorts and others utilizing disease-enriched samples, also contributed to heterogeneity and potential spectrum bias. Collectively, these factors rendered statistical pooling inappropriate and potentially misleading.

In place of a meta-analysis, a narrative synthesis was conducted. Across the five studies that reported diagnostic classification performance, AI systems consistently demonstrated strong diagnostic accuracy, with AUC values generally ranging from 0.79 to 0.98 [6,10,15,16,17]. In several domains—including anterior disc displacement detection, joint effusion identification, and MRI-based assessment of osteoarthritis—AI performance was comparable to, and in some cases numerically exceeded, that of expert human readers [6,10,16]. Notably, Yoon et al. (2023) [15] was the only included study to report independent external validation, with sustained high performance across institutions, suggesting that at least some models may be resilient to inter-institutional variation in imaging equipment and clinical environment.

Despite these promising findings, the current evidence base remains limited. All included studies were retrospective and predominantly single-center in nature, with sample sizes that remain modest for the development and validation of deep learning models. Reporting of key methodological safeguards, particularly measures to prevent data leakage and ensure unbiased model evaluation, was frequently incomplete. The absence of prospective and multi-center validation further constrains assessment of real-world clinical performance. Overall, while these findings indicate substantial potential for AI-assisted TMJ MRI interpretation, more rigorous and standardized research is required before routine clinical implementation can be recommended.

### 3.7. Results of Reporting Bias Assessment

Formal statistical assessment of reporting bias, such as funnel plot analysis, was not applicable because a meta-analysis was not conducted. Reporting bias was therefore assessed qualitatively by comparing outcomes specified in the methods with those reported in the results of the included studies. No major discrepancies were identified. However, several studies provided incomplete descriptions of AI model development and validation procedures, raising the possibility of selective or unreported analyses—a limitation commonly observed in early-stage AI diagnostic research.

### 3.8. Certainty of Evidence (GRADE)

Certainty of evidence was assessed using the GRADE framework adapted for diagnostic test accuracy studies. Overall, the certainty of evidence across TMJ MRI pathologies was judged to be low (Table 5).

For TMJ effusion, certainty was also low, reflecting serious risk of bias, single-study evidence, and limited external validation [6].

For anterior disc displacement [10,15], certainty was rated as low, downgraded due to serious risk of bias related to retrospective single-center design and unclear reporting of AI validation procedures, as well as imprecision resulting from the limited number of studies and absence of pooled estimates.

For TMJ osteoarthritis [16], certainty was rated as low, despite use of a CT-based reference standard and direct AI vs. human comparison. Downgrading was applied due to retrospective design, limited sample size, and absence of independent replication.

For disc perforation [17], certainty was judged as very low, primarily due to reliance on a single retrospective cohort without replication and limited reporting of comparator performance metrics.

Overall, although diagnostic performance metrics were high across several pathologies, methodological limitations and restricted generalizability reduced confidence in the stability of these estimates. Risk of bias was downgraded due to retrospective single-center designs and high risk in patient selection based on QUADAS-2 assessment. Imprecision reflects limited sample sizes, absence of pooled diagnostic estimates, and restricted external validation. Publication bias was suspected due to predominance of positive AI studies and lack of prospective protocol registration.

## 4. Discussion

MRI visualizes both soft and hard TMJ structures and is the gold standard for evaluating disc morphology and position, condylar form, and joint effusion. Its diagnostic accuracy is approximately 95% for disc assessment and 93% for osseous changes [18]. Despite its diagnostic reliability, increasing imaging volumes and the global shortage of radiologists have intensified the need for adjunctive technologies capable of supporting interpretation and improving workflow efficiency [19].

AI, particularly deep learning (DL) models based on convolutional neural networks (CNNs), has demonstrated substantial potential in medical image analysis through automated feature extraction and pattern recognition. In musculoskeletal imaging, AI systems have achieved high diagnostic performance across multiple joint pathologies. However, clinical implementation remains constrained by issues including dataset heterogeneity, limited external validation, and insufficient integration of broader clinical context into algorithmic decision-making [19].

The present systematic review demonstrates that AI models applied to TMJ MRI achieve high discriminative performance across diverse pathologies, including anterior disc displacement, joint effusion, osteoarthritis, and disc perforation [6,10,15,16,17]. Across included studies, reported AUC values ranged from approximately 0.79 to 0.98 [6,10,15,16,17]. In studies providing direct head-to-head comparisons, AI frequently matched expert-level overall accuracy and, in certain tasks, demonstrated higher specificity than human readers [6,10,16]. Conversely, radiologists often maintained equal or superior sensitivity [6,10,16]. These findings suggest that AI performance is comparable to experienced clinicians in structured classification tasks but does not consistently surpass expert interpretation across all diagnostic parameters.

However, interpretation of these findings must be tempered by the limited evidence base. Only five studies met the inclusion criteria, and all were retrospective, single-center investigations. This restricted number of eligible studies reflects the early and highly specialized stage of AI applications in TMJ MRI but substantially limits the robustness and generalizability of the conclusions. In accordance with our structured certainty assessment, the overall certainty of evidence was judged to be low, primarily due to methodological limitations, risk of bias in patient selection, and limited external validation. Consequently, reported performance metrics should be interpreted as preliminary estimates rather than definitive evidence of clinical equivalence.

An important observation across the included studies was the tendency for AI systems to demonstrate higher specificity but comparatively lower sensitivity than human radiologists. In the context of TMJ MRI, higher specificity is clinically advantageous, as it reduces false-positive interpretations and may prevent unnecessary additional imaging, invasive procedures, or overtreatment. This is particularly relevant in temporomandibular disorders, where imaging findings must be carefully correlated with clinical symptoms to avoid overdiagnosis. Conversely, the relatively lower sensitivity observed in some AI models raises concerns regarding the potential for missed or underdiagnosed pathology, especially in early or subtle disease stages such as minimal disc displacement or mild inflammatory changes. These complementary performance characteristics support the integration of AI as a triage or second-reader tool rather than as a standalone diagnostic system. As a triage mechanism, AI could prioritize examinations with a high likelihood of pathology, thereby improving workflow efficiency and reducing reporting delays. As a second reader, AI may enhance diagnostic confidence, reduce inter-observer variability, and provide decision support, particularly for less experienced clinicians. Such collaborative human–AI workflows align with the concept of augmented intelligence, wherein AI complements rather than replaces clinical expertise.

When contextualized within broader musculoskeletal imaging literature, similar performance patterns are observed. In wrist MRI, Lin et al. reported that a deep learning model for triangular fibrocartilage complex (TFCC) injury detection achieved higher overall accuracy and specificity than two radiologists, while one radiologist demonstrated higher sensitivity [20]. Likewise, in knee MRI studies evaluating anterior cruciate ligament (ACL) tears, deep learning models achieved diagnostic metrics approaching those of fellowship-trained radiologists, although expert readers retained marginal advantages in certain performance measures [21,22]. Across anatomical regions, AI systems appear particularly effective in achieving balanced accuracy and reducing false-positive interpretations, whereas experienced clinicians maintain strengths in sensitivity and contextual interpretation.

A consistent theme emerging from TMJ, wrist, and knee MRI studies is that AI performance often equals or exceeds that of less experienced readers and reduces inter-observer variability [6,10,15,16,17,20,21,22]. In TMJ osteoarthritis assessment, AI performance approached expert-level accuracy while clearly outperforming residents, highlighting its potential as a standardization tool [16]. Such findings suggest that AI may be especially valuable in environments with heterogeneous levels of reader expertise or high diagnostic workload.

Interpretation of AI–human comparisons must account for the heterogeneity of reference standards across the included studies. Surgical confirmation represents the highest level of diagnostic certainty, followed by CT-based criteria for osseous pathology, whereas reliance on expert MRI interpretation introduces potential subjective variability and incorporation bias. When AI models are evaluated against expert opinion rather than an independent gold standard, performance metrics may reflect agreement rather than true diagnostic accuracy. Consequently, differences in reference standards limit direct comparability across studies and may partially explain variability in reported AI–human performance.

Given the small number of eligible studies and their uniform retrospective, single-center design, the current body of evidence remains fragile. The limited sample of investigations restricts statistical robustness and increases vulnerability to publication bias. Accordingly, the certainty of evidence was rated as low, and conclusions regarding AI–human equivalence should be interpreted with caution pending prospective multicenter validation [19].

The QUADAS-2 assessment identified a high risk of bias in the patient selection domain across all included studies and an unclear risk in the index test domain. These findings have important methodological implications. Retrospective single-center designs and the use of disease-enriched cohorts may inflate estimates of diagnostic accuracy and limit generalizability. In several studies, insufficient reporting of dataset partitioning raised concerns regarding potential data leakage, particularly when image-level rather than patient-level separation was employed. Such leakage can lead to overly optimistic model performance. Additionally, the relatively small sample sizes increase the risk of model overfitting, whereby algorithms capture dataset-specific patterns that may not generalize to new populations. Only one study incorporated external validation, further restricting the assessment of model robustness across different scanners and clinical settings. Finally, the absence of standardized reporting frameworks for AI development and evaluation complicates the appraisal and comparability of studies. Future research should adhere to established guidelines such as STARD-AI and CONSORT-AI to enhance methodological transparency and reliability. The future role of AI in TMJ MRI interpretation is best conceptualized as complementary rather than substitutive. AI systems may function as second readers, quality-control mechanisms, and decision-support tools capable of enhancing diagnostic consistency, reducing inter-observer variability, and improving workflow efficiency. Additionally, AI-assisted interpretation may provide educational benefits for less experienced clinicians. Prospective multicenter validation studies, standardized head-to-head comparison designs, and outcome-based research are necessary to determine whether AI integration translates into measurable improvements in patient care.

In summary, current evidence indicates that, under controlled retrospective study conditions, AI can achieve diagnostic performance comparable to that of experienced radiologists in TMJ MRI interpretation and may enhance reproducibility and efficiency. However, AI does not consistently demonstrate superiority over expert clinicians. The evolving trajectory of AI in musculoskeletal imaging therefore supports augmentation of radiological practice rather than replacement, pending prospective validation in real-world clinical settings.

## 5. Conclusions

This systematic review demonstrates that AI systems applied to TMJ MRI achieve high diagnostic performance across multiple pathologies, including anterior disc displacement, joint effusion, osteoarthritis, and disc perforation. Reported discriminative ability was consistently strong, with AUC values ranging from 0.79 to 0.98.

In studies providing direct numerical comparison, AI performance was generally comparable to that of experienced radiologists. AI models frequently demonstrated higher specificity and comparable overall accuracy, whereas human readers often maintained higher sensitivity. Importantly, AI performance approached expert level and exceeded that of less experienced readers, suggesting potential utility in reducing inter-observer variability and supporting standardized interpretation.

At present, AI should be regarded as a complementary decision-support tool rather than a replacement for radiologists. Future multicenter, prospective studies with standardized head-to-head comparison designs are required to determine whether AI integration can translate into measurable improvements in clinical outcomes and workflow efficiency.

## Figures and Tables

**Table 1:** PICO framework.

Component	Description
P (population)	Human participants of any age and sex undergoing magnetic resonance imaging (MRI) of the temporomandibular joint (TMJ) for the diagnosis or evaluation of temporomandibular disorders (TMDs), including disc displacement or other internal derangements.
I (intervention)	Application of artificial intelligence (AI), machine learning (ML), or deep learning (DL) algorithms for TMJ MRI interpretation, classification, segmentation, or diagnostic decision support.
C (comparison)	Assessment performed by human experts, such as radiologists, oral and maxillofacial radiologists, or clinicians using conventional diagnostic criteria or manual image interpretation.
O (outcome)	Diagnostic performance of AI models compared with human experts in TMJ MRI interpretation, measured by sensitivity, specificity, accuracy, AUC, Dice coefficient, or IoU.

**Table 2:** Key characteristics of included studies.

Study	Target Condition	Participants/Joints	MRI Protocol	AI Model	Comparator
Lee et al. 2024 [6]	Effusion detection	1474 pts/2948 TMJs	3.0T; sagittal PD/T2	VGG16 CNN; ensemble; multimodal DNN	TMD specialist + radiologists
Lee et al. 2022 [10]	ADD detection	1260 pts/2520 TMJs	3.0T; sagittal oblique; PD-, T1-, and T2-weighted sequences acquired (PD used for modeling)	VGG-style CNN (fine-tune, from-scratch, freeze) + ensemble	Two expert radiologists
Yoon et al. 2023 [15]	ADD classification	502 pts (internal); 226 pts (external)	1.5T/3.0T; sagittal MRIs open/closed	ROI detection + CNN classifier with explainability	TMJ experts
Nozawa et al. 2025 [16]	TMJ-OA	118 pts/200 condyles	1.5T/3.0T; PD-weighted MRI	ResNet18, EfficientNet-b4, Inception v3, GoogLeNet	CT-based DC/TMD standard; experts and residents
Kim et al. 2021 [17]	Disc perforation	289 pts/299 TMJs	MRI + surgery	MLP; random forest	Surgical diagnosis

**Table 3:** QUADAS-2 risk-of-bias summary.

Study	Patient Selection	Index Test	Reference Standard	Flow and Timing	Applicability
Lee 2024 [6]	High	Unclear	Unclear	Low	Moderate
Lee 2022 [10]	High	Unclear	Unclear	Low	Low– Moderate
Yoon 2023 [15]	High	Unclear	Unclear	Low	Low– Moderate
Nozawa 2025 [16]	High	Unclear	Low	Low	Low– Moderate
Kim 2021 [17]	High	Unclear	Low	Low	Moderate

**Table 4:** Comparative diagnostic performance of AI models versus human experts for TMJ MRI interpretation.

Study	Target Pathology	AI Performance (Test/Validation)	Human Performance (If Reported)	Direction of Comparison
Lee et al., 2024 [6]	TMJ effusion	AUC 0.79; accuracy 0.74; sensitivity 57.4%; specificity 87.3%	Accuracy 67.7%; sensitivity 80.0%; specificity 58.2%	AI achieved higher accuracy and specificity, while humans demonstrated higher sensitivity
Lee et al., 2022 [10]	ADD detection	AUC ≈ 0.88; ensemble accuracy 0.83; sensitivity 0.82; specificity 0.85	Experts: accuracy 0.79–0.80; higher sensitivity, lower specificity	AI and experts showed comparable overall performance; AI had higher specificity, experts higher sensitivity; no statistically significant difference
Yoon et al., 2023 [15]	ADD classification	AUROC 0.985 (internal), 0.960 (external); sensitivity 0.93–0.95; specificity 0.89–0.92	Not reported as reader metrics	Excellent AI performance; no direct AI-human comparison performed
Nozawa et al., 2025 [16]	TMJ-OA	AI (ResNet18): AUC 0.91–0.93; accuracy 0.85–0.88; sensitivity 0.87, specificity 0.88; κ = 0.95 (vs. CT). Experts: AUC 0.94/0.85; accuracy 0.84; κ = 0.68. Residents: AUC 0.83/0.58; accuracy 0.71/0.56; κ = 0.27	Experts AUC ≈ 0.9, residents substantially lower	AI performance comparable to experts and clearly superior to residents
Kim et al., 2021 [17]	Disc perforation	AUC 0.94 (MLP), 0.92 (RF), Sens 96.3%	Human diagnostic metrics vs. surgery not reported	Strong AI performance versus surgical ground truth

**Table 5:** GRADE summary of certainty of evidence for AI diagnostic performance in TMJ MRI.

Outcome	No. of Studies	Risk of Bias	Inconsistency	Indirectness	Imprecision	Publication Bias	Overall Certainty
TMJ Effusion [6]	1	Serious	Not applicable	Not serious	Serious	Suspected	Low
ADD [10,15]	2	Serious	Not serious	Not serious	Serious	Suspected	Low
TMJ Osteoarthritis [16]	1	Serious	Not applicable	Not serious	Serious	Suspected	Low
Disc Perforation [17]	1	Serious	Not applicable	Not serious	Very serious	Suspected	Very Low
Overall body of evidence	5	Serious	Moderate heterogeneity	Direct population	Serious	Suspected	Low

## Data Availability

No new data were created or analyzed in this study.

## Supplementary Materials

The following supporting information can be downloaded at: https://www.mdpi.com/article/10.3390/healthcare14081066/s1, Table S1. Full Electronic Search Strategy Used Across All Databases.
