# Supplementary material for: Artificial Intelligence vs. Human Experts in Temporomandibular Joint MRI Interpretation: A Systematic Review
Source: Healthcare (Basel). 2026 Apr 17;14(8):1066. doi: 10.3390/healthcare14081066 (PMC13116614; doi:10.3390/healthcare14081066)
Supplement: Supplementary file 1 [file healthcare-14-01066-s001.zip › healthcare-4205542-supplementary.pdf]

**Supplementary Table S1**. Full Electronic Search Strategy Used Across All Databases

| Database | Search Strategy | Filters/Limitations |
|---|---|---|
| PubMed/MEDLINE | ("Temporomandibular Joint" OR TMJ) AND ("Magnetic Resonance Imaging" OR MRI) AND ("Artificial Intelligence" OR "Machine Learning" OR "Deep Learning" OR "Neural Network" OR "Convolutional Neural Network" OR CNN OR "Computer-Aided Diagnosis" OR CAD) | Humans; English language; Publication date: January 2020 - January 2026 |
| ScienceDirect | ("Temporomandibular Joint" OR TMJ) AND ("Magnetic Resonance Imaging" OR MRI) AND ("Artificial Intelligence" OR "Machine Learning" OR "Deep Learning" OR "Neural Network" OR "Convolutional Neural Network" OR CNN OR "Computer-Aided Diagnosis" OR CAD) | English language; Publication date: January 2020 - January 2026 |
| Wiley Online Library | ("Temporomandibular Joint" OR TMJ) AND ("Magnetic Resonance Imaging" OR MRI) AND ("Artificial Intelligence" OR "Machine Learning" OR "Deep Learning" OR "Neural Network" OR "Convolutional Neural Network" OR CNN OR "Computer-Aided Diagnosis" OR CAD) | English language; Publication date: January 2020 - January 2026 |
| Springer Nature Link | ("Temporomandibular Joint" OR TMJ) AND ("Magnetic Resonance Imaging" OR MRI) AND ("Artificial Intelligence" OR "Machine Learning" OR "Deep Learning" OR "Neural Network" OR "Convolutional Neural Network" OR CNN OR "Computer-Aided Diagnosis" OR CAD) | English language; Publication date: January 2020 - January 2026 |
